# Inappropriate admissions of the cardiology and orthopedics departments of a tertiary hospital in Shanghai, China

**DOI:** 10.1371/journal.pone.0208146

**Published:** 2018-12-19

**Authors:** Wenwei Liu, Suwei Yuan, Fengqin Wei, Jing Yang, Jin Ma

**Affiliations:** 1 College of Philosophy, Law and Political Science, Shanghai Normal University, Shanghai, China; 2 School of Public Health, Shanghai Jiao Tong University School of Medicine, Shanghai, China; 3 China Hospital Development Institute, Shanghai Jiao Tong University, Shanghai, China; 4 Department of Medical Administration, Shanghai Rui Jin Hospital, Shanghai, China; University of Auckland, NEW ZEALAND

## Abstract

**Objectives:**

Admission rates have increased in China, despite the fact that accessibility to primary care is improving. Hospital care could be cost-inefficient, and little is currently known about the appropriateness of admissions to tertiary hospitals in China. This study aims to measure the extent of inappropriate admissions in the cardiology and orthopedics departments of a tertiary hospital in Shanghai, to explore the factors associated with inappropriateness for each department, and to identify the causes of inappropriate admissions.

**Methods:**

The records of inpatients discharged on randomly sampled two days each week during March 2013 to February 2014 from the two departments were extracted. Two reviewers were recruited to assess the records according to the Chinese version of the Appropriateness Evaluation Protocol (C-AEP). Demographic, socio-economic, and other admissions-related variables were collected. Logistic regression analysis was adopted to determine the associated factors of inappropriateness.

**Results:**

35.0% (N = 120) of the 343 admissions and 38.7% (N = 179) of the 463 admissions of the cardiology and orthopedics departments were not justified by the C-AEP, respectively. Age (OR = 0.717), self-pay (OR = 3.752), admission via outpatient sector (OR = 5.332), and readmission (OR = 2.501) were identified as factors affecting the appropriateness of admissions in the cardiology department. Age (OR = 0.930), self-pay (OR = 2.597), admission during 12:00–17:59 (OR = 3.211), and admission via outpatient sector (OR = 7.060) were determined to be associated with appropriateness of admission in the orthopedics department. The main reason for inappropriateness was premature admission for both departments.

**Conclusions:**

The magnitude of inappropriate admissions was considerable in the departments. To improve appropriateness, the results suggest that further interventions should be focused on both external and internal factors.

## Introduction

Hospital care is normally much more expensive than other alternatives. For example, in China, the average cost of an inpatient stay was 7442.3 CNY and 2482.7 CNY of hospital and a primary health provider in 2013, respectively [1]. It is believed that part of the costs is unnecessary [2]. To avoid unnecessary hospital care and its commensurate costs, many approaches have been designed and employed worldwide, of which the Utilization Review (UR) is one of the effective methods of containing health costs. UR has been adopted by insurance organizations to make decisions regarding reimbursements according to the appropriateness of an admission or a hospital day [3]. Many appropriateness studies have been conducted to explore the prevalence, associated factors, and causes of inappropriateness in different countries and localities [4–8]. However, this kind of effort is lacking in China. Indeed, only three published studies have investigated the appropriateness of admissions in mainland China [9–11]. Moreover, these studies were either performed in a less intensive environment in township hospitals or adopted a less valid screening tool for less appropriate patient samples, e.g., patients under 16 years old [9–11].

Like many other developing countries, health reform in China has been targeted to expand the availability of health services. From 2008 to 2013, the number of health service institutions increased dramatically to above 970,000 from approximately 890,000, of which only 4,997 were hospitals, and 57,353 were primary care providers [1]. The improvement of the availability of health resources has facilitated the utilization of health services and enhanced the equity of the right to health. Normally, better perceived accessibility to primary care is related to lower admission rates [12]. However, according to the reports of the China Health Statistics Yearbook, the national admission rate has been growing steadily from 6.8% to 14.9% during the same period [1, 13], while the rates for OECD countries have been decreasing [14]. Thus, it is reasonable to consider the possibility of the existence of avoidable hospitalization.

Instead of targeting a specific part of the costs, the Global Budget (GB) program aims to contain the costs of hospital as a whole, and has been adopted by many countries [15]. To explore the feasibility of GB in China, Shanghai was designated as one of the pilot cities for hospital global budget reform in 2009. At present, the main public hospital cost containment method of the provider side in Shanghai is for the medical insurance management office of the local Municipal Human Resources and Social Security Bureau to set an upper limit through a capped GB [16]. In Shanghai, all public tertiary hospitals have been involved in the GB program since 2011 [17]. A lump sum is calculated on the basis of historical data. If extra insurance costs beyond the cap occur, the hospitals are required to share this part with the medical insurance fund. However, the piloting GB only involves basic medical insurances for urban employees and residents. For self-pay patients and patients with private insurance plans, there has been no cost-limit policy. Theoretically, the cost containment program would make the hospital more aware of the volume and structure of the services provided, but its impact on the appropriateness of utilization remains to be elucidated.

Since admission rates have been increasing despite the fact that accessibility to primary care is improving, hospital care is considered to be cost-inefficient in many cases, and little is currently known about the appropriateness of tertiary hospital use in China, it is critical to obtain a primary understanding of the appropriateness of China’s hospital utilization. This study aims to provide empirical evidence on the existence of inappropriate admission, and its associated factors and causes in a typical tertiary hospital in Shanghai, China.

## Materials and methods

### Study design

A retrospective review of the medical records of discharged patients was conducted in a tertiary teaching hospital in Shanghai. This hospital is a teaching hospital (affiliated with Shanghai Jiao Tong University) that provide both care services and health related education. The study was approved by the Academic Ethics Committee of Public Health and Nursing Research, Shanghai Jiao Tong University. The research team also signed a contract with the hospital that the data shall be used only for academic purpose. In addition, the medical records were anonymized and de-identified through the sampling and review procedure.

The cardiology department and orthopedics department of the hospital agreed to participate in our study. The two departments were involved in the hospital quality control program, which also required improvement of the writing of medical records. This hospital has 42 clinic departments and over 1,600 beds, and over 80,000 patients were discharged each year. There were 79 beds in the cardiology department and 105 beds in the orthopedics department, respectively. During March 2013 to February 2014, 3,071 and 3,915 patients were discharged from the two departments, respectively. Considering that the appropriateness of admissions could be date-dependent because some treatments might only be provided on weekdays and holidays (e.g., some surgeries are not performed on weekends) or on certain weekdays, we randomly selected two days per week during the 12 months. All of the documents of the patients admitted on the selected days were extracted from the hospital’s electronic record system. The admissions of patients who were under 16 years old or were discharged on the day of admission were excluded, as they have previously been found to be inappropriate for inclusion in such a study [9–11].

### Evaluation instrument

The admission section of the Chinese version of the Appropriateness Evaluation Protocol (C-AEP) was adopted as the screening tool in this study (see S1 Text) [18]. The original AEP was developed by Gertman and Restuccia in the U.S. in the 1980s [2], and was modified to accommodate local medical practices in many countries. The protocol is comprised of a series of diagnosis-independent descriptions of appropriate admissions for adult patients. When an admission fails to satisfy any item of the protocol, it can be justified as inappropriate. The admission part of the C-AEP contains 14 objective criteria and an override option [18]. The override option serves as the subjective part of the protocol, and it allows the reviewer(s) to use his or her own knowledge and experience to designate the appropriateness of an admission. When the override option is applied, the reviewers categorize an admission to be appropriate even if no criterion is met, or to be inappropriate even if one or more items are satisfied. Reliability and validity were tested by two groups of reviewers retrospectively in the same context of this study, and the results were presented in another paper [18]. The inter-rater agreement of the C-AEP reviewers was 0.746 (95% confidence interval [CI] 0.644–0.834), and the inter-rater agreement between C-AEP reviewers and expert reviewers was 0.678 (95% CI 0.567–0.778) [18]. Two experienced C-AEP reviewers were recruited for the study. Besides appropriateness of the admissions, an initial exploration of reasons for inappropriateness was conducted. The reviewers were required to report the reasons by using text descriptions.

The two reviewers screened the records independently. To imitate the actual review process of UR [19], the reviewers were asked to hold a meeting each day to reach a consensus on every judgement of appropriateness and reason for inappropriateness.

### Data collection

In addition to the appropriateness of the admission and its reason(s), socio-demographic and clinical information were also collected. These variables included gender, age, marital status, Hukou, employment, payment method, date of admission, admission time, admission route, readmission, service type, and comorbidity. Hukou is a residence registration system in China, and it served as the indicator for residential distance in this study. Patients were categorized as self-pay by the electronic medical record system even though some were insured by certain commercial insurance schemes. This is because, as long as the GB program only included basic medical insurances for urban employees and residents, patients with other insurance plans were the same as the self-pay patients to the hospital, and no cost concern will be involved during the process of care. Holidays include weekends and other national holidays. Admission time was divided into morning (08:00 to 11:59), afternoon (12:00 to 17:59), and night (18:00 to 07:59) according to the working shift of the hospital. The Charlson Comorbidity Index (CCI) was employed to describe the severity and complexity of a patient’s comorbidity(ies) [20]. Only the data mentioned above were collected, and the authors do not have access to information that could identify individual participants during or after data collection.

### Analysis

Continuous and categorical data were reported by median and their Inter Quartile Range (IQR) or proportions, respectively. The significance of differences was tested by the Wilcoxin Rank-Sum test and Chi-square test accordingly. The logistic regression model was adopted to explore the associated factors with inappropriate admissions. Hosmer and Lemeshow’s test was performed to examine the goodness-of-fit of the full model. The *p* value is considered significant if <0.05 in the regression analysis. Microsoft Office Excel and SPSS version 20.0 were used for data entry and analysis, respectively.

## Results

### Inappropriateness of admissions

A total of 806 records were extracted from the electronic patient history system. 343 and 463 admissions of the cardiology department and the orthopedics department were reviewed, respectively. 28.4% and 54.6% of the patients were female, and the median age was 65 (IQR = 36) and 57 (IQR = 26), respectively. 97.6% (N = 330) in the cardiology department and 86.0% (N = 394) in the orthopedics department were married. Most of the patients of the cardiology department were retired (72.5%, N = 190) and of Shanghai Hukou (74.3%, N = 249), and the percentages of the orthopedics department were 51.1% (N = 213) and 67.8% (N = 309). 42.6% (N = 146) and 47.7% (N = 211) paid by insurance, respectively. Approximately 25% of the patients were admitted on a non-working day in both departments. The patients were most frequently admitted in the morning (45.5%, N = 155) in the cardiology department, and in the afternoon (45.8%, N = 212) in the orthopedics department. Approximately half of the patients were admitted via the outpatient sector. Readmissions counted for 27.5% (N = 93) and 30.9% (N = 142) in the cardiology department and the orthopedics department, respectively. There were 88.2% (N = 300) and 85.9% (N = 397) surgical patients in cardiology and the orthopedics department, respectively. 26.2% (N = 90) and 49.2% (N = 228) of the patients had no co-morbidity when admitted, respectively.

35.0% (N = 120) and 38.7% (N = 179) of the admissions of the cardiology department and the orthopedics department were judged to be inappropriate, respectively. The override option is applied in 1.7% (N = 6) and 1.1% (N = 4) of the assessed admissions, respectively. The main reason for the use of overrides is the lack of documented information on the admission day, and the reviewers had to resort to other sources of information to decide on appropriateness (i.e., the medical treatment received on the second day). The results of the appropriate and inappropriate admissions by inpatients’ characteristics are presented (**Table 1**). No significant difference of appropriateness was found between different gender groups (*p* > 0.05). Younger patients had a higher rate of inappropriateness in both departments (*p* < 0.05). Married patients enjoyed a higher appropriateness rate than other marital status patients in the orthopedics department (*p* = 0.005), while no significant difference was found in the cardiology department (*p* = 0.269). Patients without Shanghai Hukou had significantly higher inappropriate admission rates in both departments (*p* < 0.05). The appropriateness rate of admissions significantly differed among different employment groups in the orthopedics department (*p* < 0.001). Self-pay patients had a higher possibility of being admitted inappropriately in both departments (*p* < 0.001). Whether or not the admission occurred on holidays did not affect the appropriateness of admission significantly in this study (*p* > 0.05). The rates of inappropriate admission differed significantly among different admission times in both departments (*p* < 0.001). Admissions via the outpatient sector had an inappropriateness prevalence of 53.5% and 54.7% for the cardiology department and the orthopedics department, respectively, which were higher than those for other groups (emergency and referred from other institutions). Readmitted patients were more likely to be admitted inappropriately in both departments (*p* < 0.05). There is no significant difference of appropriateness by service type for both departments. The inappropriateness rate of different CCI groups was found to differ significantly in the orthopedics department (*p* < 0.001).

**Table 1:** Inappropriate and appropriate admissions by inpatients’ characteristics.

	Cardiology department			Orthopedics department		
	Appropriate(%	Inappropriate(%)	*p*	Appropriate(%)	Inappropriate(%)	*p*
Gender	(missing = 1)					
Male	61(62.9)	36(37.1)	0.571	120(51.7)	90(42.9)	0.103
Female	162(66.1)	83(33.9)		164(64.8)	89(35.2)	
Age ^a^	67 (21)	62 (19)	0.008	61 (28)	48 (27)	0.000
Marital status	(missing = 5)			(missing = 5)		
Married	212(64.2)	118(35.8)	0.269	254(64.5)	140(35.5)	0.005
All others ^b^	7(87.5)	1(12.5)		29(45.3)	35(54.7)	
Hukou	(missing = 8)			(missing = 7)		
Non-Shanghai	43(50.0)	43(50.0)	0.001	57(38.8)	90(61.2)	0.000
Shanghai	174(69.9)	75(30.1)		223(72.2)	86(27.8)	
Employment	(missing = 81)			(missing = 46)		
Employed	47(72.3)	18(27.7)	0.643	74(49.7)	75(50.3)	0.000
Retired	133(70.0)	57(30.0)		159(41.8)	54(25.4)	
Unemployed	6(85.7)	1(14.3)		23(41.8)	32(58.2)	
Payment method						
Self-pay	78(53.4)	68(46.6)	0.000	107(48.4)	114(51.6)	0.000
Insurance	145(73.6)	52(26.4)		177(73.1)	65(26.9)	
Day of admission	(missing = 1)					
Holiday	58(78.2)	27(31.8)	0.515	77(64.2)	43(35.8)	0.514
Working day	165(64.2)	92(35.8)		207(60.3)	136(39.7)	
Admission time	(missing = 2)					
08:00–11:59	86(55.5)	69(44.5)	0.000	112(63.6)	64(36.4)	0.000
12:00–17:59	83(66.9)	41(33.1)		103(48.6)	109(51.4)	
18:00–07:59	53(85.5)	9(14.5)		69(92.0)	6(8.0)	
Admission route	(missing = 15)			(missing = 2)		
Outpatient	74(46.5)	85(53.5)	0.000	112(45.3)	135(54.7)	0.000
Emergency	137(84.6)	25(15.4)		169(80.1)	42(19.9)	
Other institutions	4(57.1)	3(42.9)		2(66.7)	1(33.3)	
Readmission	(missing = 5)			(missing = 3)		
Yes	47(50.5)	46(49.5)	0.001	74(52.1)	68(47.9)	0.008
No	173(70.6)	72(29.4)		207(65.1)	111(34.9)	
Service type	(missing = 3)			(missing = 1)		
Medical	30 (75.0)	10 (25.0)	0.216	35 (53.8)	30 (46.2)	0.216
Surgical	191 (63.7)	109 (36.3)		249 (62.7)	148 (37.3)	
CCI						
CCI = 0	55(61.1)	35(38.9)	0.600	117(91.4)	11(8.6)	0.000
CCI = 1	97(67.4)	47(32.6)		105(62.9)	62(37.1)	
CCI = 2	40(61.5)	25(38.5)		43(89.0)	5(10.4)	
CCI ≥ 3	31(70.5)	13(29.5)		19(95.0)	1(5.0)	

^a^ Continuous variable is reported as median (IQR).

^b^ “All others” refers to unmarried, divorced and widowed.

### Associated factors of inappropriateness

The associated factors of inappropriate admissions were explored by adopting logistic regression models, and the univariate and multivariate results of the cardiology department and the orthopedics department are presented in **Table 2** and **Table 3**, respectively.

**Table 2:** Univariate logistic regression analysis results.

	Cardiology department				Orthopedics department			
	OR	95% CI		*p*	OR	95% CI		*p*
		Lower	Upper			Lower	Upper	
Gender—male	0.868	0.532	1.416	0.572	1.382	0.949	2.013	0.092
Age	0.743	0.593	0.931	0.010	0.940	0.925	0.956	0.000
Marital Status–married	3.896	0.474	32.052	0.206	0.457	0.268	0.779	0.004
Hukou–non-Shanghai	2.320	1.404	3.833	0.001	4.094	2.704	6.198	0.000
Employment–employed	0.918	0.492	1.713	0.788	2.145	1.422	3.235	0.000
Employment–retired	1.195	0.650	2.198	0.566	0.308	0.204	0.465	0.000
Payment method–self-pay	2.431	1.544	3.828	0.000	2.901	1.969	4.276	0.000
Day of admission—holiday	1.198	0.710	2.021	0.499	0.850	0.552	1.308	0.460
Admission time– 08:00–11:59	2.182	1.387	3.434	0.001	0.855	0.580	1.259	0.427
Admission time– 12:00–17:59	0.880	0.553	1.402	0.592	2.736	1.861	4.024	0.000
Admission route–outpatient	5.784	3.469	9.646	0.000	4.793	3.156	7.279	0.000
Admission route–emergency	0.176	0.106	0.292	0.000	0.208	0.137	0.317	0.000
Readmission–yes	2.352	1.440	3.841	0.001	1.714	1.146	2.561	0.009
Service type–medical	0.538	0.806	3.637	0.162	0.366	0.409	1.176	0.174
CCI–CCI = 0	1.258	0.765	2.069	0.366	2.330	1.588	3.418	0.000
CCI–CCI = 1	0.836	0.532	1.314	0.438	0.903	0.611	1.336	0.610
CCI–CCI = 2	1.097	0.830	1.450	0.514	0.161	0.063	0.415	0.000

**Table 3:** Multivariate logistic regression analysis results.

Variable	β Coefficient	OR	SE	95% CI		*p*
				Lower	Upper	
**Cardiology department**						
Constant	-2.396	0.091	0.289	—		0.000
Age	-0.333	0.717	0.135	0.550	0.935	0.014
Payment method–self-pay	1.322	3.752	0.277	2.179	6.458	0.000
Admission route—outpatient	1.674	5.332	0.275	3.110	9.144	0.000
Readmission—yes	0.917	2.501	0.291	1.415	4.420	0.002
**Orthopedics department**						
Constant	0.056	1.058	0.476	—		0.906
Age	-0.073	0.930	0.011	0.910	0.951	0.000
Payment method–self-pay	0.954	2.597	0.254	1.578	4.274	0.000
Admission time– 12:00–17:59	1.167	3.211	0.254	1.950	5.286	0.000
Admission route–outpatient	1.955	7.060	0.282	4.065	12.263	0.000

In the univariate logistic regression models of the cardiology department, age (odds ratio [OR] = 0.743, 95% CI = 0.593–0.931), Hukou–non-Shanghai (OR = 2.320, 95% CI = 1.404–3.833), payment method–self-pay (OR = 2.431, 95% CI = 1.544–3.828), admission time—08:00–11:59 (OR = 2.182, 95% CI = 1.387–3.434), admission route–outpatient (OR = 5.784, 95% CI = 3.469–9.646), admission route–emergency (OR = 0.176, 95% CI = 0.106–0.292), and readmission (OR = 2.352, 95% CI = 1.440–3.841) were significantly associated with inappropriate admissions. In the full model, Hukou, and admission time were no longer associated with a higher percentage of inappropriate admissions. Age (OR = 0.717, 95% CI = 0.550–0.935), payment method–self-pay (OR = 3.752, 95% CI = 2.179–6.458), admission route–outpatient (OR = 5.332, 95% CI = 3.110–9.144), and readmission—yes (OR = 2.501, 95% CI = 1.415–4.420) were identified as factors affecting the appropriateness of admissions.

Concerning the orthopedics department, age (OR = 0.940, 95% CI = 0.925–0.956), marital status–married (OR = 0.457, 95% CI = 0.268–0.779), Hukou–non-Shanghai (OR = 4.094, 95% CI = 2.704–6.198), employment status–employed (OR = 2.145, 95% CI = 1.422–3.235), employment status–retired (OR = 0.308, 95% CI = 0.204–0.465), payment method–self-pay (OR = 2.901, 95% CI = 1.969–4.276), admission time– 12:00–17:59 (OR = 2.736, 95% CI = 1.861–4.024), admission route–outpatient (OR = 4.793, 95% CI = 3.156–7.279), admission route–emergency (OR = 0.208, 95% CI = 0.137–0.317), and readmission–yes (OR = 1.714, 95% CI = 1.146–2.561) were found to have affected inappropriateness in univariate regression models. Patients with a CCI = 0 (OR = 2.330, 95% CI = 1.588–3.418) had a significantly higher possibility of being admitted inappropriately, while the rate of patients with a CCI = 2 (OR = 0.161, 95% CI = 0.063–0.415) was significantly lower than other groups. In the full model of the multivariate regression, younger age (OR = 0.930, 95% CI = 0.910–0.951), payment method–self-pay (OR = 2.597, 95% CI = 1.578–4.274), admission time—12:00–17:59 (OR = 3.211, 95% CI = 1.950–5.286), and admission route–outpatient (OR = 7.060, 95% CI = 4.065–12.263) were associated with a higher inappropriateness rate in the orthopedics department.

### Reasons for appropriateness and inappropriateness

The reasons for inappropriate admissions were similar for these two departments (**Table 4**). Premature admission counted for 71.7% (N = 86) and 82.1% (N = 147) of the inappropriate admissions in the cardiology department and the orthopedics department, respectively. These patients needed inpatient care, but were admitted before any service could be arranged. For example, a patient was admitted on Monday, but all treatments were not started until Wednesday. 17.5% (N = 21) and 3.9% (N = 7) of the records for the cardiology department and the orthopedics department, respectively, did not provide adequate information on the justification of appropriateness in a tertiary hospital, and were deemed as inappropriate because the patients could be treated by a primary care provider or in an outpatient setting. 10.8% (N = 13) and 14.0% (N = 25) of inappropriate admissions to the cardiology department and the orthopedics department, respectively, were caused by inconvenience of transportation for the patient. These patients were mostly patients living outside of Shanghai and with mobility difficulties, and would cause high waiting cost if not admitted.

**Table 4:** Reasons for inappropriate admissions.

Reasons for inappropriateness	Cardiology department		Orthopedics department	
	n	%	n	%
Premature admission	86	71.7	147	82.1
Patient needs care at a lower level than a tertiary hospital	21	17.5	7	3.9
Inconvenience of transportation	13	10.8	25	14.0

## Discussion

Remarkably, inappropriate admission rates were 35.0% for the cardiology department and 38.7% for the orthopedics department. The rates of inappropriateness were higher than the results of previous domestic studies and the results of studies conducted in other countries and areas [9–11, 21, 22]. For example, a study conducted in Italy found that 14.8% of the admissions in the cardiology department of a second-level hospital were inappropriate [21], and another study reported that the inappropriate admissions rate was 14.6% in the orthopedics department of five hospitals in Italy [22]. Although different in terms of sampling, screening tools and numerous other aspects, the substantial rate of inappropriateness calls for caution. Our hypothesis is that the distrust of primary health service quality and the lack of a gatekeeper system may constitute the underlying causes of the higher inappropriateness rate of admissions in China. It is observed that enhancing the availability of health care can reduce hospital admission rates [12]. However, this is not currently the case in China. Although the number of primary care providers has increased, patients, particularly patients who believe that they need inpatient care, still prefer large tertiary hospitals because they lack confidence in the quality of care of primary care institutions [23]. In addition, since no compulsory gatekeeper program exists in China, patients are free to choose any health service institution, and physicians usually provide them with admission permission as soon as possible in order to retain them, or the patients could choose to go to another hospital [24].

Payment method had an impact on the appropriateness of admissions. Socio-economic factors are seldom investigated in UR projects. Previous studies have shown that lower socio-economic status was associated with a higher possibility of inappropriate admission [25, 26]. However, although the impact of payment method on hospital utilization has been discussed in numerous papers, its influence on the appropriateness of utilization has not been explored thoroughly. Our study can provide some preliminary evidence on the impact of hospital cost containment programs on admission appropriateness. In this study, the inappropriateness level of admissions of the self-pay patients was nearly four times higher than that of the insurance-pay patients in the cardiology department, and this number was approximately 2.6 in the orthopedics department (see **Table 3**). As previously mentioned, GB constitutes the main hospital cost containment method of tertiary hospitals in Shanghai. The basic mechanism was very simple: the local medical insurance management office allocates a lump sum of money to the hospital, and it is the hospital’s responsibility to determine its distribution among patients with basic medical insurances. Thus, according to target income and cost shift theories [27, 28], it is conceivable that the hospital would admit more self-pay patients inappropriately. However, by using cross-sectional data, whether or not the quantity of appropriate admissions was also affected due to inappropriate admissions remains undetermined. It is possible that the inefficiency can undermine the availability of hospital resources. It should also be noted that, in Shanghai, only the basic medical insurances for employees and residents are involved in GB. There were no cost containment measurements for other insurance plans (e.g., commercial health insurances). Therefore, from the perspective of hospitals, there was no difference between these patients and self-pay patients, and the electronic medical record system would automatically categorize them into self-pay patients. Although our conclusion that GB makes hospitals more aware of its services provided is not affected, the fact that some insured patients were categorized as self-pay patients in the self-pay group should be noted.

Admission via the outpatient sector was also identified as a common risk factor for inappropriate admissions of both clinic departments. This result was also confirmed by Poppa *et al*. and Rodríguez *et al*. [29, 30]. This finding implicated the possibility of the willingness of the responsible physicians to accelerate the process of treatment for certain patients. Some researchers implied that this phenomenon also reflected poor communication and coordination between internal sectors [30].

Older age was not justified as a risk factor for inappropriateness in this study. Nevertheless, in several studies conducted in more developed countries, less elderly patients also had lower levels of inappropriateness [21, 31]. Part of the difference is probably related to a greater clinically justified demand of medical service and a stronger willingness of staying home instead of being hospitalized in elderly patients [21]. However, since this study was targeted to investigate the admissions that had taken place, further inquiry into the hospital utilization behavior of elderly patients is requisite.

Readmission was detected as a risk factor for inappropriate admissions for the cardiology department. A potential explanation for this phenomenon is that the physicians intended to facilitate admissions of certain patients with whom they were familiar. When there is a waiting list, it is possible for physicians to expedite treatment for some patients. For example, a research conducted in Italy found that physicians intended to accelerate the diagnostic process for patients by admitting them into the hospital [30]. However, further evidence is needed to draw any conclusion here. In the orthopedics department, patients were more likely to be admitted inappropriately in the afternoon hours, and these patients might have to wait for an examination or formal physician’s order for one day or more. These minor differences of associated factors reflected that differences of clinical and managerial practice exist between the two departments.

The most common reason for inappropriate admissions was premature admission in both clinic departments. These patients were admitted before any tests, surgery, and other services were arranged. This result agrees with extant domestic studies, as well as most studies conducted in more developed countries [9–11, 21, 32–34]. The transportation issue for the patients and that the patients could be treated in less intensive settings (e.g., outpatient sector) were also pivotal reasons for inappropriate admission. The major causes of inappropriateness also underlined the critical importance of strengthening the consistency of the admission process. In addition, further qualitative studies should be performed to precisely determine the details of the delays.

Our study possesses several limitations. First, the judgement of the reviewers can only depend on clinical charts. Although the two departments in this study were in a quality control program that has specific quality requirements for record filling, the lack of solid evidence regarding the patient’s preference could still lead to one-sided conclusions. We suggest that other interested researchers use the C-AEP, in which the records are of a strict format and high quality. Alternatively, evidence also exists that the AEP can be used concurrently and prospectively [35]. Second, considering the lack of experience in using a structured tool in the same context and the retrospective nature of the study, the consensus method was employed in the reason identification process, and the results could be somewhat subjective. In order to reduce the impact of personal bias, independent judgement and group meetings were included. Particularly, every judgement of the reason for inappropriateness and the information on which it was based were required to be discussed until consensus was reached. The results also indicated that a simplified reason list of the original AEP can be developed for future studies. Third, the C-AEP only concerns services that were performed without questioning the appropriateness of the services themselves. Although the results indicated that only a few patients could be treated in less intensive settings, the possibility of unnecessary diagnostic tests, treatments, and surgeries remains. Thus, further complementary studies are needed to obtain additional evidence. Besides, only two departments voluntarily participated in this study. A larger sample size should be considered by future evaluation projects for a more comprehensive understanding of the admission appropriateness of tertiary hospitals in China.

## Conclusions

According to the C-AEP, a substantial proportion of 35.0% and 38.7% of the inpatients in the cardiology department and the orthopedics department of a tertiary hospital in Shanghai, respectively, were admitted inappropriately. The predictive characteristics of inappropriate admission were younger age, self-pay, and admitted via the outpatient sector for both departments. Patients were also at risk of being admitted inappropriately if readmitted into the cardiology department or admitted in afternoon hours in the orthopedics department. The main cause for inappropriate admission was premature admission for both departments. To reduce inappropriateness, further interventions should be focused on system factors outside of the hospital (e.g., provider cost containment programs) and organizational factors within the hospital (e.g., coordination between sectors, physicians’ behavior, etc.).

## Supporting information

S1 Text The admission section of the Chinese version of the AEP. (DOC)
