# Supplementary material for: Inappropriate admissions of the cardiology and orthopedics departments of a tertiary hospital in Shanghai, China
Source: PLoS One. 2018 Dec 19;13(12):e0208146. doi: 10.1371/journal.pone.0208146 (PMC6300262; doi:10.1371/journal.pone.0208146)
Supplement: S1 Text — (DOC) [file pone.0208146.s001.doc]

**The admission section of the Chinese version of the AEP**

**Severity of illness**

A1. Sudden onset of unconsciousness or disorientation (coma or unresponsiveness)

A2. Pulse rate: a. <50 per minute or b. >140 per minute

A3. Blood pressure:

a. Systolic <90 or >200 mmHg

b. Diastolic <60 or >120 mmHg

A4. Acute loss of sight or hearing

A5. Acute loss of ability to move body part

A6. Persistent fever >37.8℃ (by mouth) or >38.3℃ (rectally) for more than 5 days

A7. Active bleeding

A8. Severe electrolyte/blood gas abnormality (any of the following):

a. Na < 123 mEq/L; Na > 156 mEq/L

b. K < 2.5 mEq/L; K > 6.0 mEq/L

c. CO_2_ combining power (unless chronically abnormal) < 20 mEq/L; CO_2_ combining power (unless chronically abnormal) > 36 mEq/L

d. Arterial pH < 7.30 Arterial pH > 7.45

A9. EKG evidence of acute ischemia; must be suspicion of a new myocardial infarction

A10. Wound dehiscence or evisceration

**Clinical Services**

A11. Surgery or procedure scheduled that day requiring:

a. General or regional anesthesia and/or b. Equipment or facilities available only for inpatients

A12. Vital sign monitoring every 2 hours or more often (may include telemetry or bedside cardiac monitor)

A13. Chemotherapeutic agents that require continuous observation for life-threatening toxic reaction

A14. Intermittent or continuous respirator use at least every 8 hours
